# Recurrent spontaneous abortion related to balanced translocation of chromosomes: two case reports

**DOI:** 10.1186/s13256-021-02848-9

**Published:** 2021-05-24

**Authors:** Xue Wan, Linyan Li, Zulin Liu, Zhenhai Fan, Limei Yu

**Affiliations:** 1 Key Laboratory of Cell Engineering in Guizhou Province, Affiliated Hospital of Zunyi Medical University, Zunyi, 563003 Guizhou China; 2 Guizhou Provincial Sub-center for Prenatal Diagnosis, Affiliated Hospital of Zunyi Medical University, Zunyi, 563003 Guizhou China; 3 The Engineering Research Center of Zunyi Precision Medical Detection and Birth Defects Prevention and Control, Affiliated Hospital of Zunyi Medical University, Zunyi, 563003 Guizhou China

**Keywords:** Recurrent spontaneous abortion, Chromosomal abnormalities, Balanced translocations, Cytogenetics analysis, Karyotyping, Genetic counseling

## Abstract

**Background:**

Recurrent spontaneous abortion (RSA) is often idiopathic, but structural chromosomal abnormality is an important nosogenesis. Balanced translocations or inversions can lead to unbalanced gametes depending on the specific recombination and segregation patterns during meiosis. An unbalanced karyotype in the conceptus of a couple when one partner has a structural chromosomal abnormality may result in failure to implant, miscarriage, or ongoing pregnancy of a fetus with an unbalanced karyotype.

**Case presentation:**

We report two rare Han cases of RSA associated with balanced translocation of chromosomes. In case 1, a women who had had four spontaneous abortions, the karyotype was 46, XX, t (4;7) (q31;q22). In case 2, a women who had two spontaneous abortions and one stillborn fetus, the karyotype was 46, XX, t (3;15) (q12;p11.2), inv (5) (P13q13). The abnormal karyotype was not found in other chromosomes.

**Conclusions:**

It is very important that couples with more than two miscarriages be provided with chromosomal analysis. Referring couples for karyotyping will rule out or confirm possible hereditary etiology and the source of chromosomal abnormalities in recurrent miscarriages.

## Background

Chromosomal abnormalities are one of the genetic causes of reproductive abnormalities. There are two main types of chromosomal diseases, namely, those of chromosomal number and those of chromosomal structure. These chromosomal abnormalities are important causes of infertility, spontaneous abortion, recurrent abortion, teratosis, stillbirth, oligospermia or no sperm, and other abnormal fertility problems in couples of childbearing age. Other causes of recurrent miscarriage are diabetes and psychological disorders, such as depression [1, 2]. Carriers of balanced reciprocal translocations are at risk of producing gametes with unbalanced forms of the rearrangement following malsegregation of the translocation at meiosis. The carriers of reciprocal translocations can also have reproductive histories that may include infertility, recurrent miscarriage, fetal anomalies, and chromosomally abnormal offspring [3]. Should one of the partners of a couple have a balanced or unbalanced chromosomal structure abnormality, such as reciprocal or Robertsonian translocations, among others, the result may be recurrent miscarriage or the presence of physical and/or mental disorder(s) in next generation [4]. The percentage of chromosomal variations has been reported to be 5.5% in couples experiencing spontaneous abortion compared to 0.55% in the general population [5]. Individuals with balanced translocations may be phenotypically normal, but their offspring may show chromosome duplications or deletions as a result of normal meiotic segregation. Among couples with recurrent miscarriage, about 60% of translocations are reciprocal and 40% are Robertsonian (chromosomal rearrangement that in humans occurs in the five acrocentric pairs, namely chromosome 13, 14, 15, 21, and 22). Women are about twice as likely as men to have a balanced translocation [6].

Here we report our analysis of two rare cases of recurrent spontaneous abortion (RSA) in young women with balanced translocation of chromosomes. To our knowledge, this is the first report of such cases.

## Case presentation

### Cases

Case 1 was that of a 24-year-old Han woman who had a history of four spontaneous abortions, all of which occurred after more than 2 months of pregnancy. There was no history of illness, medication use, exposure to toxic/harmful substances or radiation during the pregnancy. The patient had four siblings. There were no significant abnormalities in the phenotype and intelligence of her parents and siblings. Her younger sister had given birth to a daughter whose phenotype and intelligence fell into the normal range. The other sister and her younger brother were unmarried. The mother and sister had no history of spontaneous abortion.

Case 2 was that of a 21-year-old Han woman who had been pregnant three times. During the first two pregnancies, spontaneous abortion occurred at more than 2 months of pregnancy; during the last pregnancy, B-ultrasound examination found no fetal heartbeat and fetal activity at 8 months of pregnancy. Cesarean section of the dead fetus revealed cleft lip and palate malformation. The phenotype and intelligence of the patient, her parents and a younger brother fell into the normal range. The mother had no history of spontaneous abortion.

### Methods

After the two women had provided informed consent, a peripheral venous blood sample was obtained for karyotyping. Peripheral mononuclear cells were cultured for 72 h at 37 °C in RPMI medium 1640 (Gibco, Gaithersburg, MD USA) and stimulated with phytohemagglutinin (Solarbio, Beijing, China) containing fetal bovine serum (Gibco), and then treated with colcemid (Aldrich-Sigma, St. Louis, MO, USA). G-banding of metaphase chromosomes was performed by Giemsa staining. For each patient, the numbers of chromosomes in 30 metaphase mitotic figures were counted and the karyotypes of ten cells in mitotic metaphase were analyzed by optical microscopy (OLYMPUS, Tokyo, Japan) and CytoVision software (Leica Biosystems GmbH, Nussloch, Germany). The procedure was repeated in the two cases of abnormal karyotypes. Chromosomal abnormalities were described according to the International System for Human Molecular Cytogenomic Nomenclature (ISCN; 2016).

### Results

**Fig. 1:**
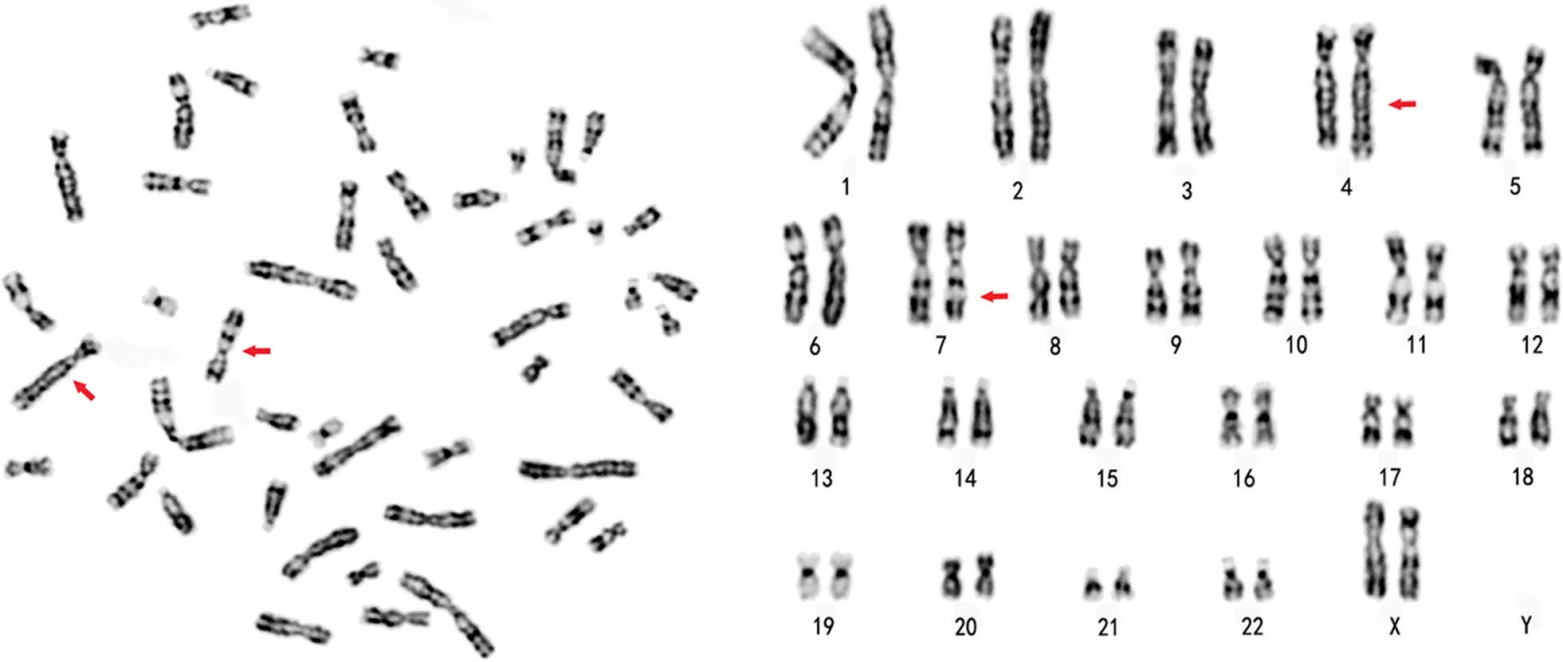
The karyogram of the case 1 patient identified by G-banding technology showing the chromosome constitution 46, XX, t (4;7)(q31;q22) mat. The arrow indicates the abnormal chromosome.

The two results of the cytogenetic examination were shown by chromosome G-banding and karyotype analysis. According to ISCN, the karyotype of the case 1 patient is 46,XX,t (4;7) (4pter → 4q31:: 7q22 → 7qter; 7pter → 7q22:: 4q31 → 4qter), as shown in Fig. 1. The abnormal karyotype was not found in other chromosomes.

**Fig. 2:**
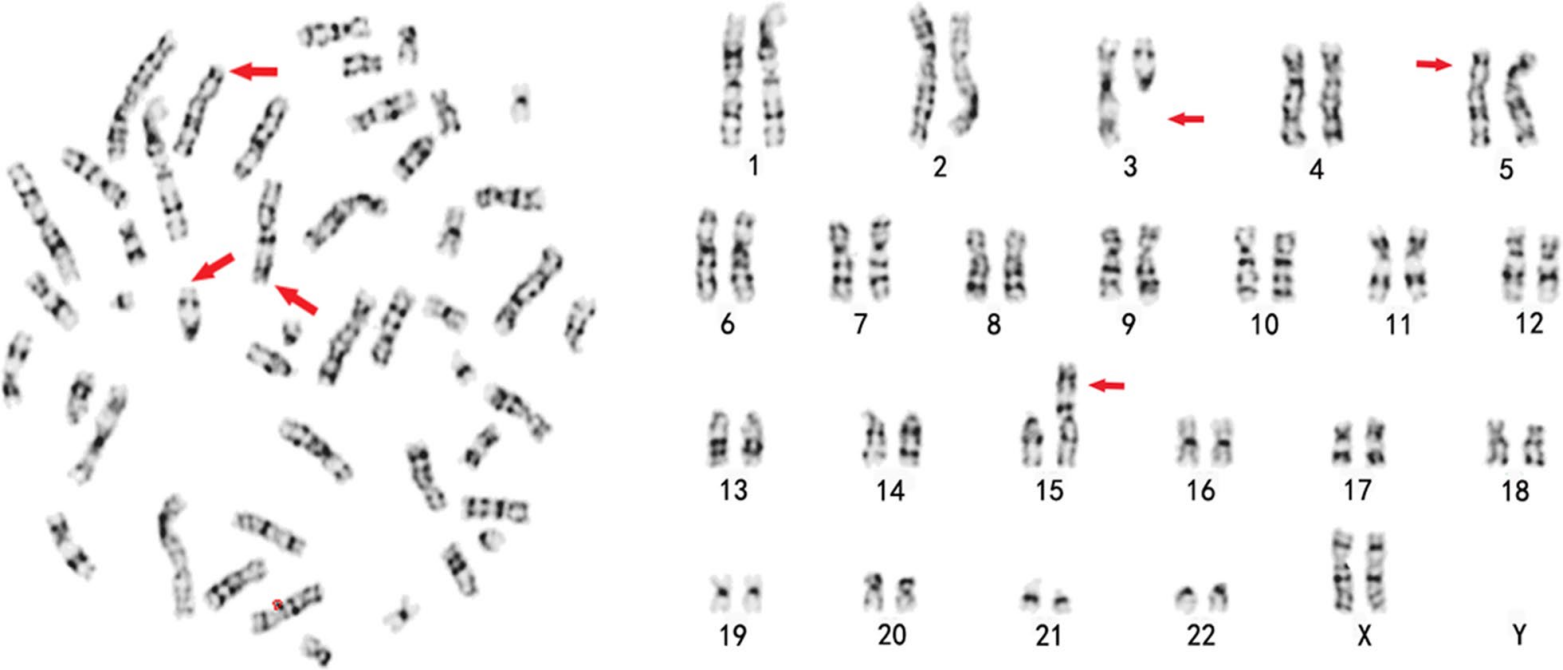
The karyotype of the case 2 patient identified by G-banding technology showing the chromosome constitution 46, XX, t (3; 15) (q12;p11.2), inv (5) (P13q13) mat. The arrow shows the abnormal karyotype.

 The karyotype of the case 2 patient is 46, XX, t (3;15) (3pter → 3q12:: 15p11.2 → 15pter; 15qter → 15p11.2:: 3q12 → 3qter), inv (5) (pter → P13:: q13 → P13:: q13 → qter), as shown in Fig. 2. The other chromosomes were normal according to G-banding.

## Discussion and conclusions

Miscarriage is the most frequent complication of pregnancy. There are several recognized causes for RSA, such as structural uterine anomalies, endocrine disorders, prothrombotic conditions (e.g., antiphospholipid syndrome), and balanced translocation involving one of the parents [7, 8]. Cytogenetic analyses have revealed a higher prevalence of chromosomal translocations in couples with recurrent miscarriage; consequently, an analysis of each translocation case is necessary to assess the risk of the woman for future miscarriage [9]. These two cases reported here involved chromosomal balanced translocations. Balanced translocation is the most common type of chromosomal structural abnormality. Although carriers of chromosomal abnormalities may have a normal phenotype in terms of body appearance and intelligence, they may have reproductive disorders, such as repeated abortion, embryo discontinuation, teratosis, abnormal semen, infertility, among others [10].

Of the many types of chromosome aberrations, equilibrium translocation is the most common. With equilibrium translocation, two non-homologous chromosomes break at the same time, exchange with each other without the centromere fragments, and form two newly derived chromosomes after conjugation. The carrier phenotype may be normal since only the relative position of the chromosome fragment is changed and there is no deletion or duplication of genetic material. No obvious phenotypic or intellectual abnormalities were found in the two cases reported here. When one or both partners of a couple have balanced translocation, unbalanced gametes may be repeatedly produced, leading to repeated abortion or a stillborn child with deformity [11]. A Turkish retrospective study reported a total of 26 chromosome abnormalities in 600 individuals, of which there were 15 cases (57.7%) of structural anomalies and 11 cases (42.3%) of numerical chromosomal aberrations. And there were five cases of balanced translocations (33.3%) and four cases of Robertsonian translocations (26.7%) in the 15 cases of structural anomalies [12]. These results show that there is a high incidence of balance heterotopias in chromosomal structural abnormalities. In our study, the two women each miscarried two or more times, consistent with an abnormal zygote in early abortion. The chromosomes involved were identified as chromosomes 4, 7, 3, and 15, whereas out of the balanced reciprocal rearrangements, chromosome 4 was involved in 26.6% of cases. Chromosome 4 has been reported to be associated with a poor obstetric history [13–15].

The karyotype of case 1 was 46, XX, t (4;7) (q31;q22). Balanced translocations on different segments of the same chromosome have been reported in both women and men, with karyotypes 46,XX,t(4;7)(q34.3;q21.3) and 46,XY,t(4;7)(q21;q11) , leading to multiple miscarriages [16, 17]. The results suggest that balanced translocation of chromosomes 4 and 7 is closely associated with recurrent miscarriage.

To the best of our knowledge, we report here for the first time an association of karyotype 46, XX, t (3;15) (q12;p11.2), inv (5) (P13q13) with recurrent abortion. It is also known that interarm inversion of chromosome is the main cause of abortion and stillbirth. It is generally believed that the length of the interarm inverted segment is related to the survival of embryos [18]. During the meiosis of parental heterozygotes, the partial deletion of a segment and partial duplication of another segment will occur if homologous pairing or exchange between the inverted chromosome and its normal homologous chromosome occurs within an inverted segment. The length of the gene duplication and of the deletion fragments and the lethal effect of the genes contained in the fragments determine the genetic effects of these two recombinant chromosomes. In general, the duration of embryo survival produced by the union of unbalanced gametes to normal chromosomal gametes depends on the size of the unbalanced fragment and the nature of the gene contained. Usually, the smaller the non-equilibrium gene segment, the more likely an infant with deformities will be born; the larger the non-equilibrium fragment, the more easily abortion occurs after the formation of the zygote. There are a few cases of balanced translocation and interarm inversion at the same time; in this scenario, the probability of giving birth to normal offspring is relatively smaller.

Recurrent abortion caused by genetic factors causes physical and psychological damage. Some studies have compared the psychological characteristics of carriers of chromosomal structural abnormalities. The results indicate that self-rating anxiety scale and self-rating depression scale scores of women with structural chromosome abnormalities were significantly higher than those of female with normal karyotypes. These two scores of women with structural chromosome abnormalities were significantly higher than those of men with structural chromosome abnormalities. These results indicate that female carriers of structural chromosome abnormalities are more vulnerable to psychological distress and require psychological support [19].

As a hereditary factor, balanced translocation is the most common occurrence among couples experienced recurrent pregnancy loss. Researchers have reported the involvement of all autosome chromosome translocations in male or female factor infertility and recurrent miscarriages [20–24]. To identify the etiology and to intervene in reproductive and prenatal diagnosis as soon as possible and to reduce the occurrence of reproductive abnormalities due to genetic factors, the pain of multiple reproductive abnormalities to families and the social burden of birth defects, we recommend that patients with a history of reproductive abnormalities should undergo routine chromosomal, high-throughput second-generation sequencing or microarray parental cytogenetic analysis before each pregnancy. Karyotype analysis remains a powerful and cheap technology and continues to have wide applications in the field of medical genetics [25]. This technology can detect chromosomal translocations or deletions and is a valuable tool in genetic counseling for infertility and abortion or intrauterine death [26].

To conclude, we describe two rare cases of balanced translocation of chromosomes associated with recurrent abortion, referring the two couples involved cases for karyotyping. Karyotype analysis will rule out or confirm the possible hereditary etiology and the source of chromosomal abnormalities in recurrent miscarriages.

## Data Availability

The datasets used and/or analyzed during the current study are available from the corresponding author on reasonable request.
