# Correction to: Recurrent spontaneous abortion related to balanced translocation of chromosomes: two case reports

**DOI:** 10.1186/s13256-021-03034-7

**Published:** 2021-08-06

**Authors:** Xue Wan, Linyan Li, Zulin Liu, Zhenhai Fan, Limei Yu

**Affiliations:** 1 Key Laboratory of Cell Engineering in Guizhou Province, Affiliated Hospital of Zunyi Medical University, Zunyi, 563003 Guizhou China; 2 Guizhou Provincial Sub-center for Prenatal Diagnosis, Affiliated Hospital of Zunyi Medical University, Zunyi, 563003 Guizhou China; 3 The Engineering Research Center of Zunyi Precision Medical Detection and Birth Defects Prevention and Control, Affiliated Hospital of Zunyi Medical University, Zunyi, 563003 Guizhou China

## Correction to: J Med Case Rep (2021) 15: 270 https://doi.org/10.1186/s13256-021-02848-9

Following publication of the original article [1], the authors identified an error in the affiliations 1–3.

Affiliation 1 and 3 should also include: Affiliated Hospital of Zunyi Medical University

Affiliation 2 indicated Zunyi Medical University Hospital while it should instead read: Affiliated Hospital of Zunyi Medical University

The affiliations have been updated in the correction article and the original article [1] has been corrected.
